# Local Convexity-Preserving *C* ^2^ Rational Cubic Spline for Convex Data

**DOI:** 10.1155/2014/391568

**Published:** 2014-03-13

**Authors:** Muhammad Abbas, Ahmad Abd Majid, Jamaludin Md. Ali

**Affiliations:** ^1^Department of Mathematics, University of Sargodha, Sargodha 40100, Pakistan; ^2^School of Mathematical Sciences, Universiti Sains Malaysia, 11800 George Town, Penang, Malaysia

## Abstract

We present the smooth and visually pleasant display of 2D data when it is convex, which is contribution towards the improvements over existing methods. This improvement can be used to get the more accurate results. An attempt has been made in order to develop the local convexity-preserving interpolant for convex data using *C* ^2^ rational cubic spline. It involves three families of shape parameters in its representation. Data dependent sufficient constraints are imposed on single shape parameter to conserve the inherited shape feature of data. Remaining two of these shape parameters are used for the modification of convex curve to get a visually pleasing curve according to industrial demand. The scheme is tested through several numerical examples, showing that the scheme is local, computationally economical, and visually pleasing.

## 1. Introduction

In computer graphics, a designer in industries needs to generate splines which can interpolate the data points in such a way that they conserve the inherited shape characteristics (positivity, monotonicity, and convexity) of data. Among the properties that the spline for curves and surfaces need to satisfy, smoothness and shape preservation of given data are mostly needed by all the designers. Convexity is a substantial shape characteristic of the data. The significance of the convexity-preserving interpolation problems in industry cannot be denied. A number of examples can be quoted in this regard, like the modelling of cars in automobile industry, aeroplane, and ship design. A crumpled curve is an unwanted characteristic. Human aesthetic sense demands convexity-preserving nice and smooth curves without wiggles [1]. Convexity should also be upheld in many applications including nonlinear programming problems occurring in engineering, telecommunication system design, approximation of functions, parameter estimation, and optimal control. The traditional cubic spline schemes have been used for quite a long time to deal with the problems of constructing smooth curves that passes through given data points. However, these splines sometimes fail to conserve the inherited shape characteristics because of unwanted oscillations that are not suitable for design purpose.

Some work [1–11, 13] on shape preservation has been published in recent years. Abbas et al. [2, 4, 5] discussed the problem of local convexity-preserving data visualization using *C* ^1^ piecewise rational cubic and bicubic function with three shape parameters. The authors derived the data dependent conditions for single shape parameter to get the convexity preserving curve and remaining shape parameters were used for the modification of convex curve to obtain a visually pleasing curve. Brodlie and Butt [6] solved the problem of shape preserving of convex data by using the cubic Hermite interpolation. The authors inserted one or two extra knots in the interval where the shape of data was not conserved. Costantini [7] solved the shape preserving of boundary valued problems using polynomial spline interpolation with arbitrary constraints. Duan et al. [12] developed rational interpolation based on function values and also discussed constrained control of the interpolating curves. They obtained conditions on function values for constraining the interpolating curves to lie above, below, or between the given straight lines. The authors assumed suitable values of parameters to obtain *C* ^2^ continuous curve and the method works for only equally spaced data.

Fiorot and Tabka [8] used *C* ^2^ cubic polynomial spline to conserve the shape of convex or monotone data. The authors obtained the values of derivative parameters by solving three systems of linear equations. Hussain et al. [9] addressed the problem of shape preserving *C* ^2^ rational cubic spline for positive and convex data. Simple data dependent constraints were derived for free parameters used in the description of rational cubic function to achieve the desired shape of the data. The scheme provided a limited freedom to designer to obtain a visually pleasing display of the data. Lamberti and Manni [10] presented and investigated the approximation order of a global *C* ^2^ shape preserving interpolating function using parametric cubic curves. The tension parameters were used to control the shape of curve. The authors derived the necessary and sufficient conditions for convexity whereas only sufficient conditions for positivity and monotonicity of data. Sarfraz et al. [11] developed a *C* ^2^ rational cubic spline with two families of free parameters for positive, monotone, and convex curve. Sufficient data dependent constraints were made for free parameters to maintain the shape of data. The scheme did not provide a liberty to designer for the refinement of positivity, monotonicity, and convexity-preserving curves.

Every developed method needs improvements or modifications to meet the required conditions. It can be used to get more accurate results. Many researchers can use new techniques to get more accurate results which are the contribution for the advancement of such results. The technique used in this paper is also a contribution to achieve the goal and has many prominent features over existing schemes.

- In this work, the degree of smoothness is *C* ^2^ continuity while, in [2, 13], it is *C* ^1^.
- In [6], the authors developed the scheme to achieve the desired shape of data by inserting extra knots between any two knots in the interval while we conserve the shape of convex data by only imposing constraints on free parameters without any extra knots.
- In [12], the authors developed schemes that work for equally spaced data while the proposed scheme works for both equally and unequally spaced data.
- The authors [14] assumed the certain function values and derivative values to control the shape of the data while, in this paper, data dependent constraints for the free parameters in the description of rational cubic function are used to achieve the required shape of the data.
- The authors [8] achieved the values of derivative parameters by solving the three systems of linear equations, which is computationally expensive as compared to methods developed in this paper where there exists only one tridiagonal system of linear equations for finding the values of derivative parameters.
- Experimental and interpolation error analysis evidence suggests that the scheme is not only local in comparison with global scheme [10] and computationally economical but also produces smoother graphical results as compared to [9, 11].
- In [11], the interpolant does not allow the designer to modify the convex curve as per industrial demands to obtain a visually pleasing curve while in this paper two out of three shape parameters are left free for designer to refine the convexity preserving curve as desired.
- The proposed curve scheme is unique in its representation and applicable equally well for the data with derivatives or without derivatives.
- The proposed scheme is not concerned with an arbitrary degree; it is a rational cubic spline in the form of cubic/quadratic and by particular setting of shape parameters; it reduces to a standard cubic Hermite spline.

This paper is organized as follows. A *C* ^2^ piecewise rational cubic function with three shape parameters is rewritten in Section 2. Local convexity-preserving rational cubic spline Interpolation is discussed in Section 3. Error estimation of interpolation is discussed in Section 5. Sufficient numerical examples and discussion are given in Section 4 to prove the worth of the scheme. The concluding remarks are presented to end the paper.

## 2. Rational Cubic Spline Function

Let {(*x*_*i*_, *f*_*i*_) : *i* = 0,1, 2,…, *n*} be the given set of data points such that *x*_0_ < *x*_1_ < *x*_2_ < ⋯<*x*_*n*_. A piecewise rational cubic function [3] with three shape parameters in each subinterval *I*_*i*_ = [*x*_*i*_, *x*_*i*+1_],  *i* = 0,1, 2,…, *n* − 1, is defined as

(1) $$\begin{array}{c} S\left(x\right)=S_{i}\left(x\right)=\frac{\sum_{i=0}^{3}{\left(1-\theta \right)}^{3-i}\theta^{i}\xi_{i}}{q_{i}\left(\theta \right)} \end{array}$$

with

(2) $$\begin{array}{c} \xi_{0}=u_{i}f_{i},\\ \xi_{1}=f_{i}\left(2u_{i}+v_{i}+w_{i}\right)+u_{i}h_{i}d_{i},\\ \xi_{2}=f_{i+1}\left(u_{i}+2v_{i}+w_{i}\right)-v_{i}h_{i}d_{i+1},\\ \xi_{3}=v_{i}f_{i+1},\\ q_{i}\left(\theta \right)={\left(1-\theta \right)}^{2}u_{i}+\theta \left(1-\theta \right)\left(w_{i}+u_{i}+v_{i}\right)+\theta^{2}v_{i}, \end{array}$$

where *h*_*i*_ = *x*_*i*+1_ − *x*_*i*_, *θ* = (*x* − *x*_*i*_)/*h*_*i*_, *θ* ∈ [0,1], and *u*_*i*_, *v*_*i*_, and *w*_*i*_ are the positive shape parameters that are used to control the shape of interpolating curve and provide the designer liberty to refine the curve as desired. Let *d*_*i*_ denote the derivative value at knots *x*_*i*_ that is used for the smoothness of curve. Let *S*′(*x*) and *S*′′(*x*) denote the first and second ordered derivatives with respect to *x*.

The following interpolatory conditions are imposed for the *C* ^2^ continuity of the piecewise rational cubic function (1):

(3) $$\begin{array}{c} S\left(x_{i}\right)=f_{i},\;\;S\left(x_{i+1}\right)=f_{i+1},\\ S^{\prime }\left(x_{i}\right)=d_{i},\;\;S^{\prime }\left(x_{i+1}\right)=d_{i+1},\\ S^{\prime \prime }\left(x_{i+}\right)=S^{\prime \prime }\left(x_{i-}\right),\;i=1,2,\ldots ,n-1 \end{array}$$

with

(4) S′′(xi+)  =2((ui+2vi+wi)Δi−vidi+1−(ui+vi+wi)di)hiui,S′′(xi−)  =(2(ui−1di−1+(ui−1+vi−1+wi−1)di  −(2ui−1+vi−1+wi−1)Δi−1))  ×(hi−1vi−1)−1.

From (3), the *C* ^2^ interpolating conditions produce the following system of linear equations:

(5) $$\begin{array}{c} \alpha_{i}d_{i-1}+\delta_{i}d_{i}+\gamma_{i}d_{i+1}=\lambda_{i} \end{array}$$

with

(6) αi=uiui−1hi,δi=hiui(ui−1+vi−1+wi−1)+hi−1vi−1(ui+vi+wi),γi=vivi−1hi−1,λi=vi−1hi−1(ui+2vi+wi)Δi+uihi(2ui−1+vi−1+wi−1)Δi−1,

where Δ_*i*_ = (*f*_*i*+1_ − *f*_*i*_)/*h*_*i*_. The *C* ^2^ piecewise rational cubic function (1) is reformulated after using (2) as

(7) $$\begin{array}{c} S\left(x_{i}\right)=\frac{p_{i}\left(\theta \right)}{q_{i}\left(\theta \right)} \end{array}$$

with

(8) pi(θ)=uifi(1−θ)3+(fi(2ui+vi+wi)+uihidi)×θ(1−θ)2+(fi+1(ui+2vi+wi)−vihidi+1)×θ2(1−θ)+vifi+1θ3,qi(θ)=(1−θ)2ui+θ(1−θ)(wi+ui+vi)+θ2vi.

**Remark 1 (see [3])**

The system of linear equations defined in (5) is a strictly tridiagonal and has a unique solution for the derivatives parameters *d*_*i*_, *i* = 1,2,…, *n* − 1 for all *u*_*i*_, *v*_*i*_ > 0 and *w*_*i*_ ≥ 0. Moreover, it is efficient to apply LU decomposition method to solve the system for the values of derivatives parameters *d*_*i*_′*s*.

**Remark 2 (see [3])**

To make the rational cubic function smoother, *C* ^2^ continuity is applied at each knot. The system (5) involves *n* − 1 linear equations while unknown derivative values are *n* + 1. So, two more equations are required for unique solution. For this, we impose end conditions at end knots as

(9) $$\begin{array}{c} S^{\prime }\left(x_{0}\right)=d_{0},\;\;S^{\prime }\left(x_{n}\right)=d_{n}. \end{array}$$

**Remark 3 (see [3])**

For the values of shape parameters set as *u*_*i*_ = 1, *v*_*i*_ = 1, and *w*_*i*_ = 0 in each subinterval *I*_*i*_ = [*x*_*i*_, *x*_*i*+1_], *i* = 0,1, 2,…, *n* − 1, the rational cubic function reduces to standard cubic Hermite spline [15].

## 3. Local Convexity-Preserving Rational Cubic Spline Interpolation

In this section, we discuss the solution of convexity-preserving problem by using *C* ^2^ rational cubic function with three shape parameters. For this problem, we impose appropriate constraints on single shape parameter to conserve the shape of convex data. This requires some mathematical arguments so that the required shape of data is achieved.

Let {(*x*_*i*_, *f*_*i*_) : *i* = 0,1, 2,…, *n*} be the given convex data set. This data set is said to be convex if

(10) $$\begin{array}{c} \;\Delta_{i}<\Delta_{i+1},\;i=0,1,\ldots ,n-2. \end{array}$$

In similar way, it is concave if

(11) $$\begin{array}{c} \Delta_{i}>\Delta_{i+1},\;i=0,1,\ldots ,n-2. \end{array}$$

For strictly convex curves, necessary condition for derivative parameters to obtain the smoothness is

(12) $$\begin{array}{c} d_{0}<\Delta_{0}<d_{1}<\cdots <\Delta_{i-1}<d_{i}<\Delta_{i}<\cdots <d_{n}. \end{array}$$

For concave data we have

(13) $$\begin{array}{c} d_{0}>\Delta_{0}>d_{1}>\cdots >\Delta_{i-1}>d_{i}>\Delta_{i}>\cdots >d_{n}. \end{array}$$

Necessary conditions for convexity are

(14) $$\begin{array}{c} u_{i}>0,\;\;v_{i}>0,\;\;w_{i}\geq 0,\\ \left(\Delta_{i}-d_{i}\right)>0,\\ \left(d_{i+1}-\Delta_{i}\right)>0. \end{array}$$

Now the *C* ^2^ rational cubic function *S*_*i*_(*x*), defined in (7), is convex if and only if *S*_*i*_′′(*x*) > 0 such that

(15) $$\begin{array}{c} S_{i}^{\prime \prime }\left(x\right)=\frac{\sum_{k=0}^{5}{\left(1-\theta \right)}^{5-k}\theta^{k}C_{k,i}}{h_{i}{\left(q_{i}\left(\theta \right)\right)}^{3}} \end{array}$$

with

(16) C0,i=2ui2{(ui+vi)(Δi−di)+wi(Δi−di)−vi(di+1−Δi)},C1,i=C0,i+2ui2(5vi(Δi−di)+vidi),C2,i=C0,i+6ui(vi2(di+1−Δi)+2uivi(Δi−di)),C3,i=C5,i+6vi(ui2(Δi−di)+2uivi(di+1−Δi)),C4,i=C5,i+2vi2(5ui(di+1−Δi)+uidi+1),C5,i=2vi2{(ui+vi)(di+1−Δi)   +wi(di+1−Δi)−ui(Δi−di)},Si′′(x)>0 if  ∑k=05(1−θ)5−kθkCk,i>0,  hi(qi(θ))3>0.

Since *u*_*i*_ > 0, *v*_*i*_ > 0 and *w*_*i*_ ≥ 0, it follows that *h*_*i*_(*q*_*i*_(*θ*))^3^ > 0:

(17) $$\begin{array}{c} \overset{5}{\underset{k=0}{\sum }}{\left(1-\theta \right)}^{5-k}\theta^{k}C_{k,i}>0\;\text{if}\;\;C_{k,i}>0,\;\;k=0,1,2,3,4,5. \end{array}$$

Hence *C*_*k*,*i*_ > 0, *k* = 0,1, 2,3, 4,5, if the shape parameters satisfy the following constraints:

(18) $$\begin{array}{c} u_{i}>0,\;\;v_{i}>0,\\ w_{i}>\max\left\{0,\frac{v_{i}\left(d_{i+1}-\Delta_{i}\right)}{\left(\Delta_{i}-d_{i}\right)},\frac{u_{i}\left(\Delta_{i}-d_{i}\right)}{\left(d_{i+1}-\Delta_{i}\right)}\right\}. \end{array}$$

The above constraints can be rewritten as

(19) $$\begin{array}{c} u_{i}>0,\;\;v_{i}>0,\\ w_{i}=\alpha_{i}+\max\left\{0,\frac{v_{i}\left(d_{i+1}-\Delta_{i}\right)}{\left(\Delta_{i}-d_{i}\right)},\frac{u_{i}\left(\Delta_{i}-d_{i}\right)}{\left(d_{i+1}-\Delta_{i}\right)}\right\},\;\alpha_{i}>0. \end{array}$$

The above discussion can be summarized as follows.

**Theorem 4**

The rational cubic function (7) conserves the *C* ^2^ convex curve of strictly convex data in each subinterval [*x*_*i*_, *x*_*i*+1_] if and only if the shape parameters *u*_*i*_, *v*_*i*_, and *w*_*i*_ satisfy (19).

## 4. Numerical Examples and Discussion

In this section, the efficiency of the proposed convexity-preserving scheme through several numerical examples is presented. A comparison of *C* ^2^ scheme with PCHIP (piecewise cubic Hermite interpolating polynomial, Built-in MATLAB program) and cubic Hermite spline scheme is also part of this section.

**Example 1**

**Figure 1:**
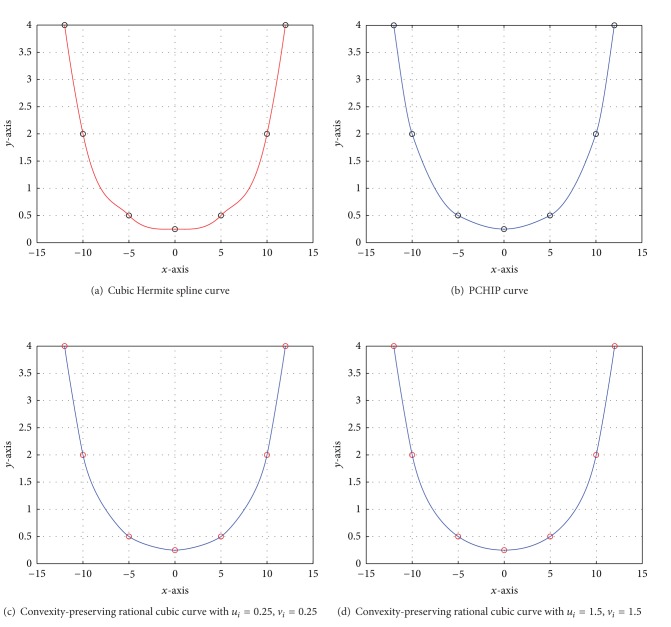
Convexity- and nonconvexity-preserving curves.

A convex data set is taken in Table 1 which is borrowed from [4].  Figure 1(a) is drawn by cubic Hermite spline scheme [15] that does not conserve the local convexity through given convex data.  Figure 1(b) is generated by PCHIP that does not look smooth because the function has only ability to remove the undulations in shape preserving curves. On the other hand, Figures 1(c) and 1(d) are generated by developed local convexity-preserving *C* ^2^ rational cubic function with different values of parameters *u*_*i*_ and *v*_*i*_. The effect of shape parameters can be seen by noting the difference in smoothness of the curves in Figures 1(b), 1(c), and 1(d). Numerical results of Figure 1(d) are determined from developed scheme shown in Table 2.

**Example 2**

**Figure 2:**
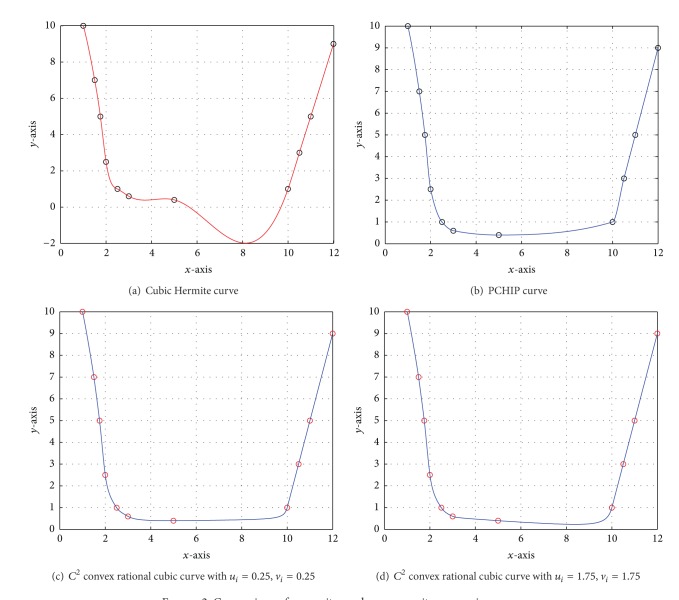
Comparison of convexity- and nonconvexity-preserving curves.

A 2D convex data set is taken in Table 3 which is borrowed from [9].  Figure 2(a) is generated by cubic Hermite spline scheme [15] that does not maintain the convexity of given data.  Figure 2(b) is produced by PCHIP to conserve the convexity of convex data but it looks tight at some data points. Figures 2(c) and 2(d) are generated by convexity-preserving rational cubic interpolant developed in Section 3. A comparison of convexity-preserving curve through convex data in these figures depicts the flaw of the cubic Hermite scheme and tightness of PCHIP.  Figure 2(d) looks more pleasant and smooth as compared to Figure 2(b). Numerical results of Figure 2(d) are determined by developed convexity-preserving *C* ^2^ rational cubic spline scheme shown in Table 4.

**Example 3**

**Figure 3:**
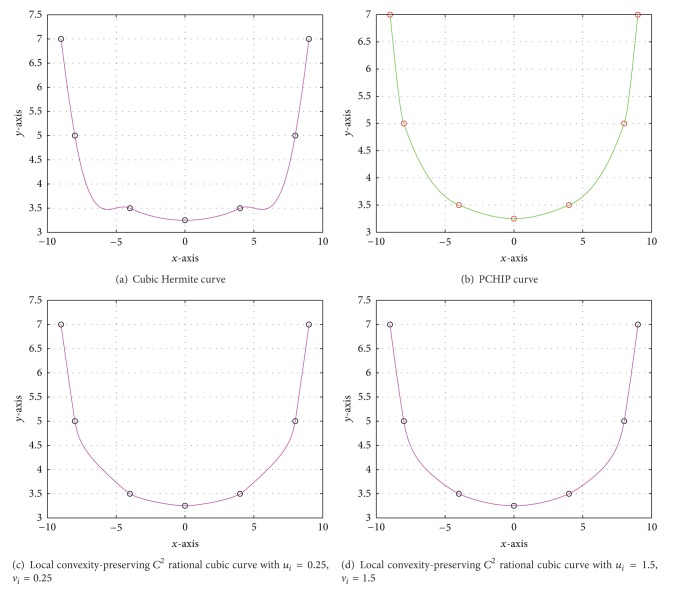
Convex and nonconvex curves through given convex data set.

The cubic Hermite spline scheme [15] and PCHIP have been used to draw Figures 3(a) and 3(b), respectively, through convex data given in Table 5 which is borrowed from [4]. The efficiency of the scheme developed in Section 3 can be seen in Figures 3(c) and 3(d). A remarkable difference in the smoothness with a pleasant graphical view is visible in these figures drawn by PCHIP and proposed rational cubic scheme due to the freedom granted to the designer on the values of shape parameters.  Table 6 demonstrates the numerical results computed from the proposed scheme of Figure 3(d).

**Example 4**

**Figure 4:**
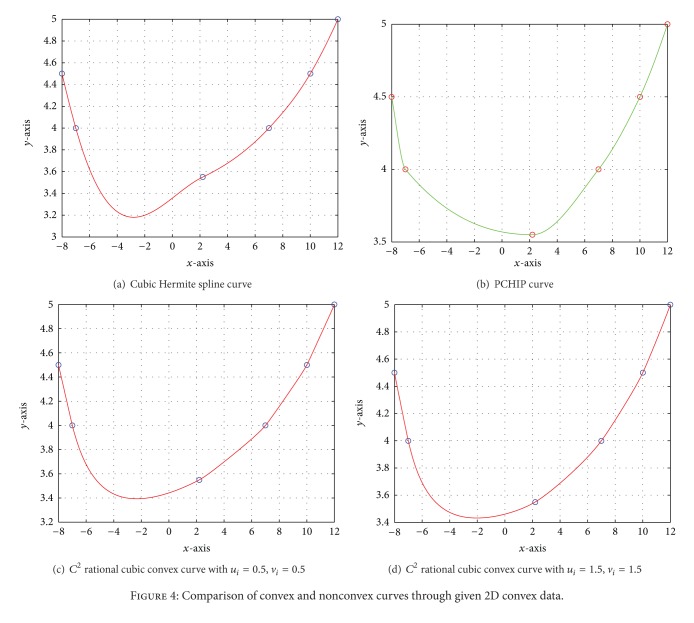
Comparison of convex and nonconvex curves through given 2D convex data.

A convex data set is taken in Table 7. A nonconvex curve from this given data is drawn in Figure 4(a) by cubic Hermite scheme [15].  Figure 4(b) is produced by using PCHIP to conserve the shape of curve but the visual model looks tight as compared to proposed rational model, whereas Figures 4(c) and 4(d) are generated by convexity-preserving rational cubic function developed in Section 3. A comparison of convexity-preserving curve in these figures depicts the flaw of the cubic Hermite spline scheme and tightness of PCHIP.  Figure 4(d) looks more pleasant and smooth as compared to Figures 4(c) and 4(b) due to different values of shape parameters. The numerical results computed from proposed scheme of Figure 4(d) are shown in Table 8.

## 5. Error Estimation

In this section, the error of interpolation is calculated by using the following Theorem 5 which was developed by Abbas et al. in [3] at some fixed values of free shape parameters *u*_*i*_, *v*_*i*_ and various values of constrained parameter *w*_*i*_. Here, we take different values of shape parameters than [3] for the effectiveness of proposed interpolant.

**Theorem 5 (see [3])**

The error of interpolating rational cubic function (7), for *f*(*x*) ∈ *C* ^3^[*x*_0_, *x*_*n*_], in each subinterval *I*_*i*_ = [*x*_*i*_, *x*_*i*+1_] is

(20) |f(x)−Si(x)|≤12||f(3)(τ)||∫xixi+1|Rx[(x−τ)+2]|dτ=||f(3)(τ)||cici=max⁡0≤θ≤1ξ(ui,vi,wi,θ),

where

(21) $$\begin{array}{c} \xi \left(u_{i},v_{i},w_{i},\theta \right)=\{\begin{array}{cc} \max\xi_{1}\left(u_{i},v_{i},w_{i},\theta \right) & 0\leq \theta \leq \theta^{*}\\ \max\xi_{2}\left(u_{i},v_{i},w_{i},\theta \right) & \theta^{*}\leq \theta \leq 1\\ \max\xi_{3}\left(u_{i},v_{i},w_{i},\theta \right) & 0\leq \theta \leq 1, \end{array} \end{array}$$

where *ξ*_1_(*u*_*i*_, *v*_*i*_, *w*_*i*_), *ξ*_2_(*u*_*i*_, *v*_*i*_, *w*_*i*_), and *ξ*_3_(*u*_*i*_, *v*_*i*_, *w*_*i*_) are given in [3].

**Theorem 6 (see [3])**

For any given positive values of shape parameters *u*_*i*_, *v*_*i*_, and *w*_*i*_, the optimal error *c*_*i*_ in Theorem 5 satisfies 0 < *c*_*i*_ ≤ 0.0640.

**Proof**

See immediately Tables 9, 10, 11, and 12.

## 6. Concluding Remarks

A *C* ^2^ rational cubic function has been developed in this paper for the smooth and attractive display of convex data. Three shape parameters were utilized for the description of function to conserve the shape of convex data. Simple data dependent sufficient constraints were derived for single shape parameter to insure convexity. Remaining two shape parameters have provided freedom to designer to modify the shape of the curve by simply adjusting the values of the shape parameters. No extra knots were inserted in the interval where the interpolant loses the convexity. The values of derivative parameters were achieved by solving the single system of linear equations in comparison with [8]; there exist three systems of linear equations for finding these values which is computationally expensive and time-consuming process. The proposed scheme is not only *C* ^2^, smoother, local, and computationally economical but also visually pleasing as compared to schemes developed in [9, 11]. The proposed scheme works for both equally and unequally spaced data while the schemes developed in [12, 14] work for only equally spaced data.

## Figures and Tables

**Table 1:** 2D convex data set.

*i*	1	2	3	4	5	6	7
*x*_*i*_	−12	−10	−5	0	5	10	12
*y*_*i*_	4	2	0.5	0.25	0.5	2	4

**Table 2:** Numerical results of Figure 1(d).

*i*	1	2	3	4	5	6	7
*d*_*i*_	−1.2	−0.7743	−0.1143	0	0.1143	0.7743	1.2
Δ_*i*_	−1.0	−0.3	−0.05	0.05	0.3	1.0	—
*u*_*i*_	1.5	1.5	1.5	1.5	1.5	1.5	—
*v*_*i*_	1.5	1.5	1.5	1.5	1.5	1.5	—
*w*_*i*_	1.1165	1.0776	0.0598	0.0598	1.0776	1.1165	—

**Table 3:** A convex data set.

*i*	*x*_*i*_	*y*_*i*_
1	1.0	10.0
2	1.5	7.0
3	1.75	5.0
4	2.0	2.5
5	2.5	1.0
6	3.0	0.6
7	5.0	0.4
8	10.0	1.0
9	10.5	3.0
10	11	5.0
11	12	9.0

**Table 4:** Numerical results of Figure 2(d).

*i*	*d*_*i*_	Δ_*i*_	*u*_*i*_	*v*_*i*_	*w*_*i*_
1	−4.6667	−6.0	1.75	1.75	1.8958
2	−7.2864	−8.0	1.75	1.75	2.3586
3	−9.1942	−10.0	1.75	1.75	0.001
4	−7.8005	−3.0	1.75	1.75	1.0835
5	−1.5996	−0.8	1.75	1.75	0.2874
6	−0.5057	−0.1	1.75	1.75	10.063
7	−0.0737	0.12	1.75	1.75	14.339
8	3.8472	4.0	1.75	1.75	167.23
9	4.0102	4.0	1.75	1.75	155.37
10	3.9991	4.0	1.75	1.75	1.600*e*7
11	4.0	—	—	—	—

**Table 5:** A convex data set.

*i*	1	2	3	4	5	6	7
*x*_*i*_	−9	−8	−4	0	4	8	9
*y*_*i*_	7	5	3.5	3.25	3.5	5	7

**Table 6:** Numerical results of Figure 3(d).

*i*	1	2	3	4	5	6	7
*d*_*i*_	−2.325	−1.644	−0.132	0	0.132	1.644	2.325
Δ_*i*_	−2	−0.375	−0.062	0.062	0.375	2	—
*u*_*i*_	1.5	1.5	1.5	1.5	1.5	1.5	—
*v*_*i*_	1.5	1.5	1.5	1.5	1.5	1.5	—
*w*_*i*_	5.503	7.902	1.181	1.181	7.902	5.503	—

**Table 7:** 2D convex data set.

*i*	1	2	3	4	5	6
*x*_*i*_	−8	−7	2.2	7	10	12
*y*_*i*_	4.5	4	3.55	4	4.5	5

**Table 8:** Numerical results of Figure 4(d).

*i*	1	2	3	4	5	6
*d*_*i*_	−0.544	−0.452	0.060	0.136	0.215	0.283
Δ_*i*_	−0.5	−0.048	0.093	0.166	0.25	—
*u*_*i*_	1.5	1.5	1.5	1.5	1.5	—
*v*_*i*_	1.5	1.5	1.5	1.5	1.5	—
*w*_*i*_	5.108	3.620	1.811	3.532	3.638	—

**Table 9:** Values of *c*_*i*_ for several values of shape parameters *u*_*i*_, *v*_*i*_, and *w*_*i*_.

*i*	*u*_*i*_	*v*_*i*_	*w*_*i*_	*c*_*i*_
1	0.01	0.01	0.01	0.0180
2	0.01	0.01	0.1	0.0458
3	0.01	0.01	0.6	0.0600
4	0.01	0.01	0.9	0.0613
5	0.01	0.01	1.6	0.0625
6	0.01	0.01	6.0	0.0637
7	0.01	0.01	12.0	0.0638
8	0.01	0.01	115.0	0.0639
9	0.01	0.01	515.0	0.0640
10	0.01	0.01	700.0	0.0640
11	0.01	0.01	1000.0	0.0640
12	0.01	0.01	1515.0	0.0640
13	0.01	0.01	2000.0	0.0640
14	0.01	0.01	20000.0	0.0640

**Table 10:** Values of *c*_*i*_ for different values of shape parameters *u*_*i*_, *v*_*i*_, and *w*_*i*_.

*i*	*u*_*i*_	*v*_*i*_	*w*_*i*_	*c*_*i*_
1	1.50	1.50	1.5	0.0104
2	1.50	1.50	0.1	0.0109
3	1.50	1.50	0.6	0.0135
4	1.50	1.50	0.9	0.0151
5	1.50	1.50	1.6	0.0185
6	1.50	1.50	6.0	0.0331
7	1.50	1.50	12.0	0.0429
8	1.50	1.50	115.0	0.0608
9	1.50	1.50	515.0	0.0633
10	1.50	1.50	700.0	0.0635
11	1.50	1.50	1000.0	0.0637
12	1.50	1.50	1515.0	0.0639
13	1.50	1.50	2000.0	0.0640
14	1.50	1.50	20000.0	0.0640

**Table 11:** Values of *c*_*i*_ with different values of shape parameters *u*_*i*_, *v*_*i*_, and *w*_*i*_.

*i*	*u*_*i*_	*v*_*i*_	*w*_*i*_	*c*_*i*_
1	50.50	50.50	1.5	0.0104
2	50.50	50.50	0.1	0.0104
3	50.50	50.50	0.6	0.0105
4	50.50	50.50	0.9	0.0105
5	50.50	50.50	1.6	0.0106
6	50.50	50.50	6.0	0.0113
7	50.50	50.50	12.0	0.0122
8	50.50	50.50	115.0	0.0257
9	50.50	50.50	515.0	0.0460
10	50.50	50.50	700.0	0.0496
11	50.50	50.50	1000.0	0.0532
12	50.50	50.50	1515.0	0.0564
13	50.50	50.50	2000.0	0.0581
14	50.50	50.50	20000.0	0.0634

**Table 12:** Values of *c*_*i*_ for various values of shape parameters *u*_*i*_, *v*_*i*_, and *w*_*i*_.

*i*	*u*_*i*_	*v*_*i*_	*w*_*i*_	*c*_*i*_
1	200.0	200.0	1.5	0.0104
2	200.0	200.0	0.1	0.0104
3	200.0	200.0	0.6	0.0104
4	200.0	200.0	0.9	0.0104
5	200.0	200.0	1.6	0.0104
6	200.0	200.0	6.0	0.0106
7	200.0	200.0	12.0	0.0108
8	200.0	200.0	115.0	0.0149
9	200.0	200.0	515.0	0.0272
10	200.0	200.0	700.0	0.0313
11	200.0	200.0	1000.0	0.0363
12	200.0	200.0	1515.0	0.0421
13	200.0	200.0	2000.0	0.0458
14	200.0	200.0	20000.0	0.0616
